# Two Decades of Negative Thermal Expansion Research: Where Do We Stand?

**DOI:** 10.3390/ma5061125

**Published:** 2012-06-20

**Authors:** Cora Lind

**Affiliations:** Department of Chemistry, the University of Toledo, Toledo, OH 43606, USA; E-Mail: cora.lind@utoledo.edu; Tel.: +1-419-530-1505; Fax: +1-419-530-4033

**Keywords:** negative thermal expansion, mechanisms, composites, challenges

## Abstract

Negative thermal expansion (NTE) materials have become a rapidly growing area of research over the past two decades. The initial discovery of materials displaying NTE over a large temperature range, combined with elucidation of the mechanism behind this unusual property, was followed by predictions that these materials will find use in various applications through controlled thermal expansion composites. While some patents have been filed and devices built, a number of obstacles have prevented the widespread implementation of NTE materials to date. This paper reviews NTE materials that contract due to transverse atomic vibrations, their potential for use in controlled thermal expansion composites, and known problems that could interfere with such applications.

## 1. Introduction

**Figure 1:**
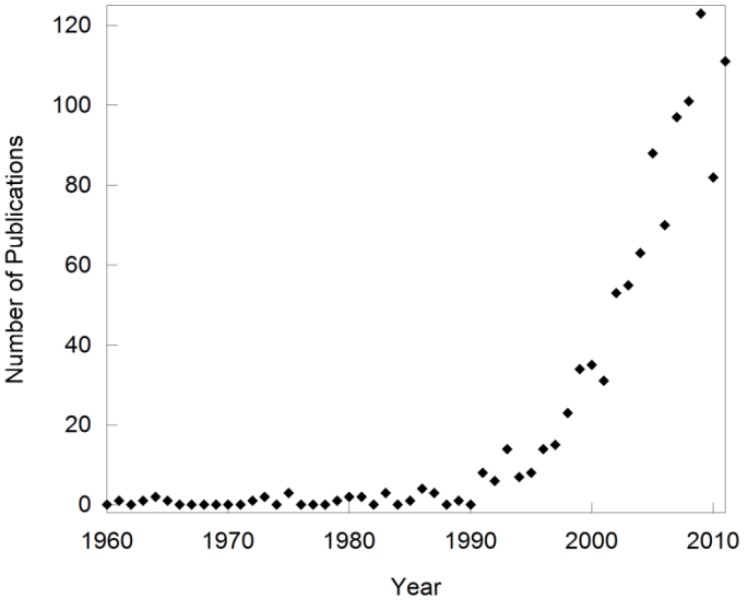
Number of publications per year based on a Web of Science search for “negative thermal expansion”. Note that early publications related to the field are missing as they did not use NTE as a keyword.

Over the past two decades, the field of negative thermal expansion (NTE) has rapidly expanded [1,2,3,4,5,6,7,8,9,10,11,12,13,14,15,16,17,18,19,20,21,22,23,24]. This is evident when tracking the number of publications on “negative thermal expansion” over the past 50 years (Figure 1), and the fact that special issues of journals have been devoted to the topic [25]. Such sudden growth of new research fields is often related to the discovery of a new phenomenon or a new class of compounds. However, the first observation of compounds that contract upon heating dates back several hundred years to the discovery of the “density anomaly of water”. Shrinkage of a solid was first documented by Scheel in 1907 for quartz and vitreous silica at low temperatures [26,27], and additional reports of materials that contract over various temperature ranges appeared in the literature throughout the years. This included research on lithium aluminum silicates (LAS) in the 1950s [28,29], and the discovery of the sodium zirconate phosphate (NZP) family in the 1980s [30,31,32,33]. These materials can show either positive or negative volume expansion depending on composition, as contraction is observed along only one or two of the crystallographic axes. They were usually referred to as “low expansion ceramics” instead of “NTE materials”, and the term “NTE” was used only sporadically between the 1960s and the 1990s. Notably, the expansion behavior of ZrW_2_O_8_ [34], which has since become one of the most researched NTE compounds and is often used as the key representative of NTE, was measured over the temperature range 323 to 973 K in 1968 [35]. However, this behavior remained a peculiarity until the mid 1990’s, when Sleight’s group could show that the NTE behavior of several families of compounds was intimately related to their crystal structures [1,2,3,5]. This included the first observation of inherently isotropic NTE over a large temperature range in cubic ZrV_2_O_7_ [1] and ZrW_2_O_8_ [3]. Theoretical and experimental studies soon established sophisticated models that can be used to explain this unusual behavior for a number of framework compounds [6,8,11,12,15,16,21,36,37,38,39,40,41,42,43,44]. This opened up the targeted synthesis of new NTE compositions, and established NTE as a specialized field of research. Several new families of materials in which NTE is caused by different mechanisms have been discovered as well, but they are outside the scope of this review. Compounds belonging to the LAS and NZP families will not be discussed in detail either, as they were already well established as low expansion ceramics by the time NTE became a separate field of research.

The potential uses of NTE materials in controlled thermal expansion composites were readily recognized, and possible applications ranging from fiber optics coatings, electronics and mirror substrates to tooth fillings were proposed [9,45,46,47,48]. However, some limitations of different NTE materials became quickly apparent, one of which relates to the fact that many NTE compounds contain transition metals, which would increase product cost. In addition, problems with stability under processing and use conditions, and incompatibilities with other composite components were encountered [49,50,51,52]. These challenges have become active areas of research, and efforts are directed at the discovery of new NTE materials, improvements of properties of existing materials, modification of particles to achieve compatibility, and establishing processing conditions for formation of homogeneous composites.

## 2. Negative Thermal Expansion Due to Transverse Vibrations

**Figure 2:**
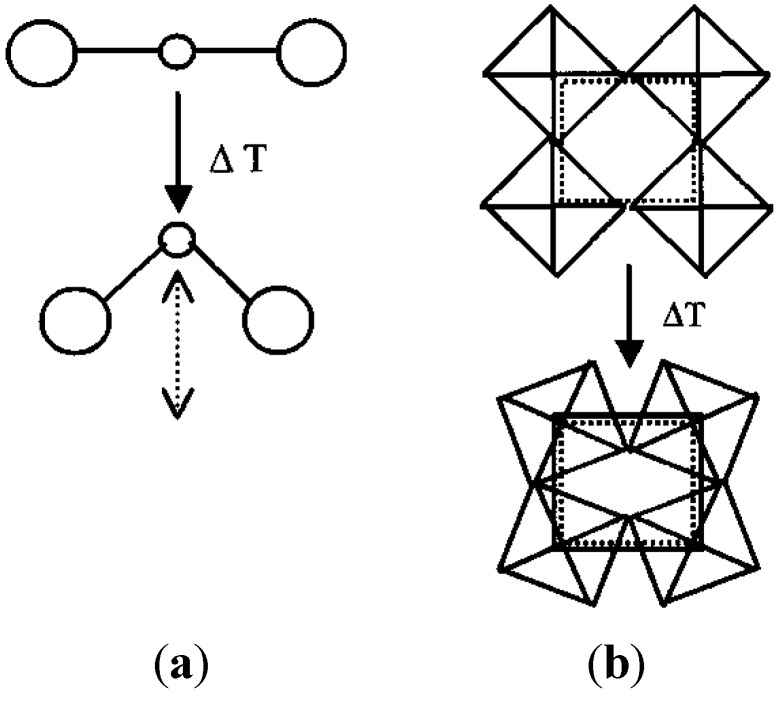
Schematics of vibrational modes leading to NTE: (**a**) Transverse vibrational motion of an oxygen atom in a M-O-M linkage causing a decrease of the metal-metal distance; (**b**) cooperative rocking of polyhedra causing a decrease in average metal-metal distances.

The expansion behavior of most NTE materials that were known or discovered in the 1990’s can be explained based on their crystal structure. These compounds are composed of rigid MO_4_ tetrahedra and/or MO_6_ octahedra, which are connected by corner-sharing oxygen atoms. Due to the corner-sharing nature of the frameworks, the polyhedra can undergo concerted tilting or rocking motions when transverse vibrations of the corner-sharing oxygen atoms are excited. For approximately linear M-O-M linkages, this process leads to a reduction of second-nearest-neighbor distances, and can result in linear or volume NTE (Figure 2). This mechanism operates in materials belonging to the zirconium tungstate family [3,53,54], scandium tungstate family [5,23], zirconium vanadate family [1], a number of zeolites and aluminum phosphates [24,55,56], Prussian blue analogs [57,58], and a few other materials [59]. Early theoretical models treated the polyhedra as rigid units, and referred to the concerted lattice vibrations as “rigid unit modes”, or RUMs [37,59,60,61,62]. 

The RUM model can adequately describe the NTE behavior observed in ZrW_2_O_8_ and some zeolites, however, the polyhedra in the ZrV_2_O_7_ and Sc_2_W_3_O_12_ families have been found to distort. These distortions have resulted in more varied values for expansion coefficients for the same structural family depending on the size and rigidity of the polyhedra. Similar behavior has also been observed in some cyanides, where the CN linkages undergo vibrations that lead to a shortening of metal-metal distances. Due to the greater flexibility of the two-atom linker, the expansion behavior can vary widely from strong NTE to positive expansion. In some cyanide frameworks, other mechanisms also contribute to NTE behavior.

The transverse vibration mechanism can also be expressed in terms of low energy librational phonon modes with large, negative Grüneisen parameters (γ). The presence of such modes has been proven experimentally through specific heat [8,39,63], phonon density of states [6,10,39], and total neutron scattering studies [12,64,65] Because the overall expansion behavior of a compound depends on the relative contributions from all phonons, not all materials with low energy phonon modes with negative γ_i_ values will exhibit NTE behavior. A feature necessary for the occurrence of NTE is the presence of low energy phonons with negative γ_i_ values, and a phonon gap that separates these modes from the high energy phonons also present in the structure [21].

### 2.1. ZrW_2_O_8_ Family

**Figure 3:**
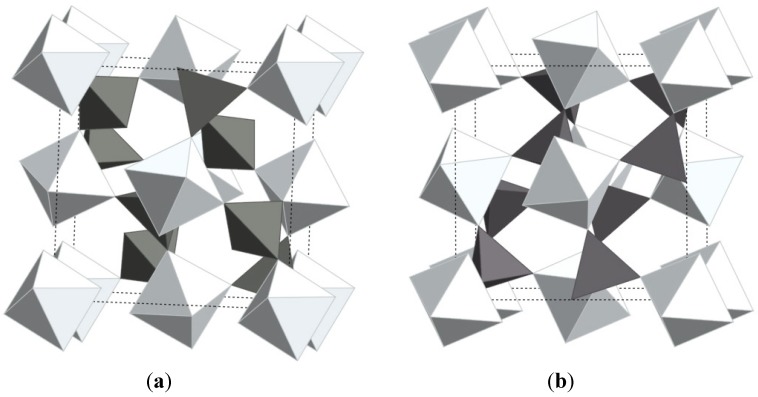
Crystal structures of (**a**) α-ZrW_2_O_8_; (**b**) ZrV_2_O_7_ (ideal high temperature structure); bright: ZrO_6_ octahedra; dark: WO_4_/VO_4_ tetrahedra. The structures only differ in the orientation and connectivity of the polyhedra.

While a number of materials have been found to contract upon heating, the compound zirconium tungstate has become almost synonymous with the expression NTE. ZrW_2_O_8_ was first discovered in 1959 by Graham [34] and its unusual expansion behavior was documented by Martinek and Hummel in 1968 [35] At that time, the strong contraction was regarded as equally detrimental as strong positive expansion, and the search for zero expansion materials moved on to different materials. In 1995, Auray solved the crystal structure of ZrW_2_O_8_ [66], and in 1996, Sleight’s group showed that the structure is responsible for the strong NTE behavior observed from 0.3 to 1050 K [3]. The material is thermodynamically stable between 1378 and 1508 K, but can be quenched and remains metastable up to 1050 K. The structure is composed of corner-sharing ZrO_6_ octahedra and WO_4_ tetrahedra, with each ZrO_6_ connected to six WO_4_ units, while each tetrahedron is connected to only three octahedra, leaving one terminal oxygen. The WO_4_ units are oriented along the body diagonal of the cubic cell, and can be described as W_2_O_8_ units with one 4-coordinated tungsten, and a tungsten with 4+1 coordination due to a long range contact with an oxygen from the neighboring tungsten (Figure 3a).

The material’s contraction is inherently isotropic due to its cubic structure, with α_l_ values of −9.1 × 10^−6^ K^−1^ below 350 K, and −5.0 × 10^−6^ K^−1^ above 450 K. The magnitude of expansion changes due to an order-disorder phase transition at 448 K (α-ZrW_2_O_8_ to β-ZrW_2_O_8_), but the cubic symmetry is preserved (space groups P2_1_3 and Pa3, respectively). The transition involves a reorientation of the WO_4_ tetrahedra, and causes a discontinuity in the otherwise linear expansion behavior. The ZrW_2_O_8_ structure supports rigid unit modes, which are responsible for the strong NTE over a large temperature range. Phonon density of states measurements revealed significant contributions from very low energy modes with negative Grüneisen parameters, and the presence of a gap between low and high energy phonons [6,8,39].

#### 2.1.1. Substitution of ZrW_2_O_8_

Both metal sites in the ZrW_2_O_8_ family can be substituted. Shortly after the pivotal ZrW_2_O_8_ paper, NTE compounds prepared by substitution of Hf on the Zr site and Mo on the W site were reported [53,54,67]. The HfW_2_O_8_ analog shows essentially identical expansion behavior with respect to the magnitude of α, and only a small increase in the temperature of the order-disorder phase transition (T_trs_ = 463 K) is observed. In contrast, ZrMo_2_O_8_ and HfMo_2_O_8_ do not undergo a transition to the ordered P2_1_3 structure, but adopt space group Pa3 at all temperatures. A transition from dynamic to static oxygen disorder occurs at low temperatures, which increases the magnitude of NTE (α_l_ −8 × 10^−6^ K^−1^ below 200 K, and −5 × 10^−6^ K^−1^ from 200 to 600 K) [68]. Early research suggested that both molybdates were metastable at all temperatures and could only be obtained by dehydration and topotactic recrystallization from a hydrated precursor (AM_2_O_7_(OH)_2_·2(H_2_O); A = Zr, Hf; M = Mo, W) [54,69,70]. However, recent *in situ* diffraction experiments provide evidence that cubic ZrMo_2_O_8_ may be stable in sealed tubes above 1350 K. The disappearance of diffraction peaks at higher temperatures was interpreted as melting, as little to no formation of ZrO_2_ was observed at 1460 K, and the cubic phase recrystallized during quenching experiments [71]. The sealed environment is necessary to prevent evaporation of MoO_3_, which becomes volatile above 1000 K under atmospheric pressure.

The complete range of Zr_1−x_Hf_x_W_2−y_Mo_y_O_8_ solid solutions (0 ≤ x ≤ 1; 0 ≤ y ≤ 2) can be synthesized either from AM_2_O_7_(OH)_2_·2(H_2_O) precursors or by solid-state methods [72,73]. This is not surprising, as Zr^4+^ and Hf^4+^ (0.72 Å and 0.71 Å in octahedral coordination) and Mo^6+^ and W^6+^ (0.55 Å and 0.56 Å in tetrahedral coordination) have very similar ionic radii to each other. Like for the pure tungstates, hafnium substitution does not significantly change the expansion and phase transition behavior of the solid solutions. In contrast, the molybdenum content strongly influences formation of the ordered P2_1_3 phase. For compositions with more than 50% tungsten, the order-disorder (α to β) transition is observed, and the temperature varies linearly with composition. ZrMoWO_8_ remains in the Pa3 structure when rapidly cooled, but formation of the P2_1_3 polymorph was observed at about 270 K upon slow cooling [74]. No ordered phase has been reported for y > 1, although it is possible that ordering was not detected for some compositions due to slow kinetics. While ZrW_2_O_8_ adopts a fully ordered structure at low temperatures, some local disordered regions remain even to the lowest temperatures for ZrMoWO_8_.

Substitution of ZrW_2_O_8_ by elements other than Hf and Mo is also possible, although solubility is limited in all cases even when metals with identical charges are chosen. The highest substitution levels have been achieved with Sn^4+^ in Zr_0.7_Sn_0.3_W_2_O_8_ [75]. The solubility of Ti^4+^ is limited to about 5% due to its much smaller ionic radius (octahedrally coordinated Ti^4+^: 0.61 Å), which induces lattice strain [76] In both cases, a reduction in the α-β phase transition temperature was observed (400 K for Zr_0.7_Sn_0.3_W_2_O_8_ and 405 K for Zr_0.95_Ti_0.05_W_2_O_8_, respectively). The expansion coefficients show limited dependence on composition, and fall around −10 × 10^−6^ K^−1^ for materials in the α-phase, and −5 × 10^−6^ K^−1^ for materials in the β-phase.

Aliovalent ions can also be incorporated into the ZrW_2_O_8_ structure. Substitution of the Zr/Hf site by a number of trivalent ions (Sc, Y, In, Eu, Er, Yb, Lu) has been reported [77,78,79,80,81]. These systems show limited solubility, ranging from 1.6% for Eu^3+^ to 5% for Yb^3+^. However, even small amounts can lead to significant changes in T_trs_. For example, 4% substitution lowers T_trs_ to 390 K for Y^3+^, 380 K for In^3+^, and 360 K for Sc^3+^ [80]. This clearly indicates that the trivalent substituents introduce disorder into the ZrW_2_O_8_ framework. Even at very low temperatures, only partial ordering is observed, similar to ZrMoWO_8_. The phase transition temperature can be correlated to the normalized saturated order parameter η. All trivalent cations investigated are larger than Zr^4+^ or Hf^4+^. In addition, an oxygen vacancy is created for every two A^3+^ cations, which is evident from the decrease of the lattice constant with increasing substitution by the larger cations. This results in a distortion of the AO_6_ octahedra, which in turn act as a local, spherical disturbance on the bonded MO_4_ tetrahedra. Yamamura et al. analyzed the anisotropic peak broadening observed for Sc, In and Y substituted ZrW_2_O_8_, and quantified the size of the distorted region as 1.3 to 1.7 nm, which is equivalent to 8 to 12 WO_4_ units [80]. The expansion behavior in the disordered high temperature phase was identical to β-ZrW_2_O_8_, regardless of identity and quantity of substituent, while slightly less negative expansion was observed with increasing A^3+^ content in the α-phase.

The only example of aliovalent substitution of the M site to date is the compound ZrV_0.2_W_1.8_O_7.9_ [82,83]. This material was reported to crystallize in space group Pa3, which is also adopted by both β-ZrW_2_O_8_ and ZrV_2_O_7_. The main difference between these structures lies in the fact that the V_2_O_7_ groups in ZrV_2_O_7_ are truly centrosymmetric, while the W_2_O_8_ groups are not, requiring equal amounts of opposite orientations to give an average centrosymmetric structure. A later publication on the same composition assigned space group P2_1_3 at room temperature, and reported a transition to the Pa3 polymorph between 358 and 400 K. Interestingly, the β-phase expansion is less negative (−1.6 × 10^−6^ K^−1^) than for most compositions (−5 × 10^−6^ K^−1^), while the α-phase expansion is identical to ZrW_2_O_8_. The solubility limit of vanadium in ZrW_2_O_8_ has not yet been determined.

#### 2.1.2. High Pressure Behavior

Open framework compounds are prone to undergo pressure-induced phase transitions. As the preparation and use of composites is likely to expose NTE fillers to pressure, their application requires investigation of their high pressure behavior. *In situ* experiments and measurements on samples recovered from high pressure have been reported for ZrW_2_O_8_ [4,7,84,85,86,87], HfW_2_O_8_ [63,88,89], ZrMo_2_O_8_ [54,90,91,92], and HfMo_2_O_8_ [90], but no solid solutions have been studied under pressure to date.

**Figure 4:**
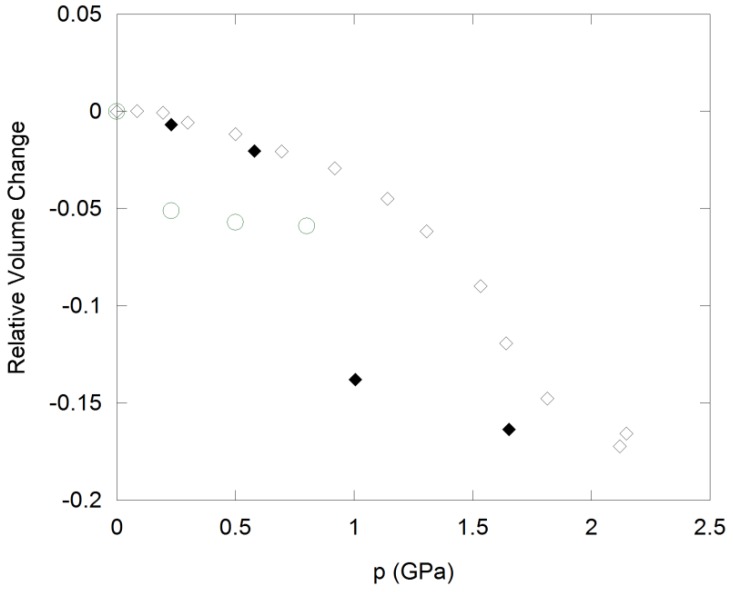
Compressibility of cubic ZrW_2_O_8_ (circles) and cubic ZrMo_2_O_8_ (diamonds) under hydrostatic conditions. Open symbols: Data collected upon compression; solid symbols: Data collected upon decompression.

Cubic ZrW_2_O_8_ undergoes an irreversible phase transition to γ-ZrW_2_O_8_ at 0.2 to 0.3 GPa (Figure 4), which is accompanied by a 5% decrease in volume per formula unit. The structure of this phase is closely related to the α-polymorph, and involves a reorientation of one third of the W_2_O_8_ units, which results in tripling of one cell axis and lowering of the symmetry to the orthorhombic system [93]. γ-ZrW_2_O_8_ can be quenched to ambient conditions, and converts back to the cubic structure upon heating to 390 K. It shows weak NTE below 225 K, and positive expansion at higher temperatures. Further compression of ZrW_2_O_8_ results in pressure-induced amorphization between 1.9 and 2.4 GPa [7]. The amorphous phase is ~25% denser than the cubic starting material, and can be retained upon decompression. To recrystallize α-ZrW_2_O_8_ at ambient pressure, heating to 923 K is necessary. Both pressure-induced phase transitions are accompanied by an increase in tungsten coordination: The orthorhombic unit cell contains one 4-coordinated W, four W atoms in 4 + 1 coordination, and one W with a 5 + 1 environment. Further changes in W coordination towards more centrosymmetric environments have been observed in PDF and XANES/EXAFS experiments during amorphization [87,94]. Zr K-edge EXAFS data on samples recovered after compression also suggest that the Zr coordination number may increase to 7 at higher pressures. These observations are consistent with a mechanism of amorphization that involves disordering of existing structural polyhedra, which leads to formation of additional bonds that crosslink the polyhedra and increase the metal coordination.

HfW_2_O_8_ shows very similar behavior to ZrW_2_O_8_, except that the transition to the γ-polymorph occurs at higher pressure (0.63 GPa) and shows sluggish kinetics [89]. Full conversion is only achieved after 24 h at 0.63 GPa, while no transformation occurs even after 11 d at 0.52 GPa. Amorphization is observed at ~2 GPa. Both γ- and amorphous HfW_2_O_8_ are metastable under ambient conditions. The orthorhombic material can be converted back to the cubic phase by heating to 360 K. No reports are available on the recrystallization of amorphous HfW_2_O_8_.

ZrMo_2_O_8_ and HfMo_2_O_8_ also undergo pressure-induced amorphization, and EXAFS/XANES studies on ZrMo_2_O_8_ suggest that the amorphous phase possesses similar local structures to ZrW_2_O_8_ [92]. The amorphous materials cannot be reconverted to the cubic structures. Instead, the more stable trigonal and monoclinic AM_2_O_8_ polymorphs are formed during heating at low and high pressure, respectively. Application of non-hydrostatic pressure results in amorphization at 0.5 to 1.5 GPa, while crystallinity is retained under hydrostatic conditions up to 3.0 GPa [90]. In hydrostatic environments, a crystalline-to-crystalline transition at 0.7–2.0 GPa precedes amorphization. This transformation is accompanied by a 10–11% decrease in cell volume (Figure 4), suggesting that the still unknown structure of the high pressure polymorph is different from γ-ZrW_2_O_8_. The data could be fitted in a pseudo-cubic cell, although subtle peak splitting indicative of symmetry lowering was evident. The transition is reversible upon decompression, although considerable hysteresis is observed, with the original cubic phases reforming below 1.0 GPa.

### 2.2. ZrV_2_O_7_ Family

Negative thermal expansion in the zirconium vanadate family was first reported in the mid 90’s [1]. ZrV_2_O_7_ is thermodynamically stable up to ~1075 K, where decomposition into binary oxides is observed. Like ZrW_2_O_8_, ZrV_2_O_7_ adopts a cubic structure, making its expansion behavior inherently isotropic. The ideal ZrV_2_O_7­_ structure is closely related to the rocksalt structure, with Zr^4+^ as the cation, and (V_2_O_7_)^4−^ as the anion. The V_2_O_7_ groups order along the threefold rotation axis, lowering the overall symmetry to Pa3, and constraining the V-O-V bond angles to be linear on average, although significant displacements of the oxygen atoms from the inversion centers have been reported. The cations are octahedrally coordinated by oxygen, and the octahedra and tetrahedra form a corner-sharing 3D network (Figure 3b). This structural arrangement can give rise to transverse vibrations of corner-sharing oxygen atoms, which may result in NTE. In contrast to ZrW_2_O_8_, such vibrational modes always involve distortions of the polyhedra. The vibrations can thus not be described by the RUM model, but may be regarded as “quasi-rigid unit modes”, or qRUMs [59]. ZrV_2_O_7_ shows strong NTE with α_l_ values between −7 and −10 × 10^−6^ K^−1^ above 375 K, but undergoes phase transitions to an incommensurate phase, and to an ordered cubic 3 × 3 × 3 superstructure in space group Pa3 upon cooling to 375 and 350 K, respectively. In the room temperature superstructure, 2/3 of the V-O-V linkages are no longer constrained to be linear on average by symmetry, and the expansion coefficient becomes positive [95,96,97].

#### 2.2.1. Substitution of ZrV_2_O_7_

The ZrV_2_O_7_ structure can accommodate a wide range of tetra- and pentavalent ions. For M = P, the A^4+^ cation can be Zr, Hf, Ti, U, Th, Pu, Np, Mo, W, Ce, Pb, Sn, Ge or Si [1], while for the vanadates only the Zr and Hf compounds [1], and for the arsenates only the Zr and Th compounds are known [98,99]. For a number of years, all compounds in the AM_2_O_7_ family were believed to adopt the ideal ZrV_2_O_7_ structure in space group Pa3 at high temperatures, and to transform to the cubic 3 × 3 × 3 superstructure at lower temperatures. This view has been challenged over the past decade. While SiP_2_O_7_, TiP_2_O_7_, and HfV_2_O_7_ indeed adopt a cubic 3 × 3 × 3 superstructure, NMR and high resolution synchrotron studies have shown that the symmetry is lower for many materials. For example, SnP_2_O_7_ and GeP_2_O_7_ were found to be monoclinic [100,101,102], CeP_2_O_7_ and AnP_2_O_7_ (An = U, Th, Pu, Np) were reported as triclinic [103,104], and ZrP_2_O_7_ and HfP_2_O_7_ exhibit an orthorhombic distortion [105,106]. The structure of the latter compounds was solved independently from synchrotron single crystal and powder diffraction data by the Birkedal and Evans groups, and consists of a 136 atom unit cell in space group Pbca. All P-O-P bond angles are significantly smaller than 180°, eliminating the possibility of transverse oxygen vibrations that could lead to a unit cell contraction [105,106]. However, high resolution studies of AnP_2_O_7_ have shown that NTE can be observed at high temperature in materials with bent P-O-P units [104]. Interestingly, SnP_2_O_7_ does not adopt the ideal ZrV_2_O_7_ structure at all, but undergoes a series of different distortions up to its decomposition temperature. Insufficient data are available to unambiguously address whether other AM_2_O_7_ compounds undergo symmetry-lowering distortions.

The magnitude of expansion of AM_2_O_7_ compounds depends on the identity of the A and M cations, which determine the size of the polyhedra. Larger polyhedra can accommodate the distortions required for transverse oxygen vibrations more easily, and as a result, most phosphates show positive expansion at all temperatures, while the vanadates show strong NTE in the high temperature phase. Phosphates with large A^4+^ cations (CeP_2_O_7_, AnP_2_O_7_) show a change from positive to negative expansion with increasing temperature.

The phase transition temperature for the ideal ZrV_2_O_7_ can be suppressed by formation of solid solutions Zr_1−x_Hf_x_V_2−y_P_y_O_7_ [1] While substitution of Hf for Zr has a limited effect on the phase transition temperature, the incorporation of P on the M site strongly influences the phase transition behavior. For small amounts of mixing, the transition temperatures for both phase changes are lowered, and for values of 0.4 < y < 1.6, the materials adopt the high temperature ZrV_2_O_7_ structure at room temperature. Expansion coefficients range from small positive to strongly negative depending on composition.

Reports of substitution of the ZrV_2_O_7_ structure by aliovalent cations are rare, and are limited to formation of A_0.5_^3+^A’_0.5_^5+^P_2_O_7_ (A = Bi, Nb, Nd, Eu, Al, Fe, Ga, In, Y; A’ = Nb, Ta) [107,108], Nb_0.05_Y_0.05_Zr_0.9_P_2−x_V_x_O_7_ [109], and ZrV_2-x_Mo_x_O_7+x/2_ (0 ≤ x ≤ 0.8) [110]. Positive expansion was observed at all temperatures for the phosphates. The incorporation of Y^3+^ and Nb^5+^ into ZrP_2-x_V_x_O_7_ reduced the positive expansion in the low temperature superstructure, but did not significantly alter the phase transition temperature and magnitude of NTE in the high temperature phase. Similarly to V-substituted ZrW_2_O_8_, the Mo-substituted ZrV_2_O_7_ crystallizes in space group Pa3, and the structure is closely related to the ZrMo_2_O_8_ and ZrV_2_O_7_ parent structures. The expansion behavior determined for a single crystal with 25% Mo substitution is similar to that of ZrV_2_O_7_.

#### 2.2.2. High Pressure Behavior

High pressure studies in the ZrV_2_O_7_ family have been limited to TiP_2_O_7_, ZrP_2_O_7_, CeP_2_O_7_, ZrV_2_O_7_ and HfV_2_O_7_ [103,111,112,113,114]. Interestingly, *in situ* diffraction experiments show no evidence for pressure-induced phase transitions or amorphization for TiP_2_O_7_ and ZrP_2_O_7_, both of which are positive thermal expansion compounds. Raman studies suggest that subtle structural changes could be occurring upon compression, but cell volumes extracted from X-ray data showed smooth compression up to 40 and 20 GPa, respectively [111].

In contrast, CeP_2_O_7_, ZrV_2_O_7_ and HfV_2_O_7_ undergo phase transitions to crystalline high pressure phases at 0.65 GPa, 1.6 GPa and 3.7 GPa. Peak splitting indicates lowering of the cubic symmetry in all cases. For CeP_2_O_7_, a second crystalline high pressure phase is observed above 5 GPa, while pressure-induced amorphization occurs above 4.0 GPa for ZrV_2_O_7_. HfV_2_O_7_ also progressively amorphizes, but traces of crystallinity are still observed at 42 GPa. Both transitions in CeP_2_O_7_ are reversible upon decompression. Raman studies on the vanadates suggest that amorphization is irreversible, whereas the crystalline high pressure phase reverts back to the ambient pressure polymorph upon decompression.

### 2.3. Sc_2_W_3_O_12_ Family

**Figure 5:**
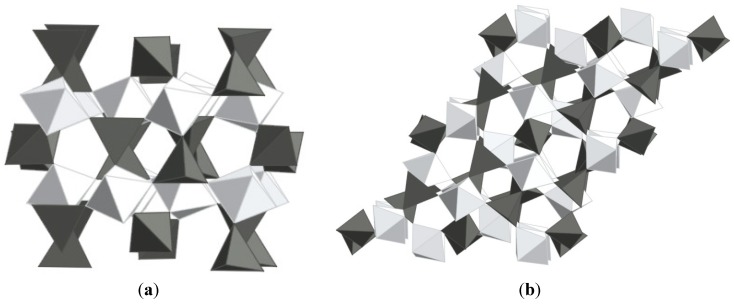
Crystal structures of (**a**) orthorhombic; (**b**) monoclinic Al_2_Mo_3_O_12_; bright: AlO_6_ octahedra; dark: MoO_4_ tetrahedra.

The scandium tungstate family has also attracted a lot of attention, as it offers a wide range of possible compositions, and the potential to tune the expansion behavior of the resulting compounds [5,23]. Sc_2_W_3_O_12_ is thermodynamically stable over a wide temperature range, allowing straightforward preparation by traditional ceramic methods. Unlike ZrW_2_O_8_ and ZrV_2_O_7_, it does not adopt a cubic structure, but crystallizes in the orthorhombic space group Pnca [115]. This gives rise to anisotropic expansion. The crystal structure is composed of a corner-sharing network of ScO_6_ octahedra and WO_4_ tetrahedra (Figure 5a). Negative volume expansion is observed from 10 to 1300 K [115,116], and an average α_l_ value of −2.2 × 10^−6^ K^−1^ was determined from variable diffraction data for the range 50–450 K. Dilatometry on ceramic bars gave significantly more negative values of −6 to −11 × 10^−6^ K^−1^ which was attributed to the presence of microcracks in the bars combined with the anisotropic expansion of the three unit cell axes, where the a and c axis contract, while the b axis expands. Detailed structural analysis showed that there were only small changes in bond distances and angles within the polyhedra as a function of temperature, but large amplitudes of vibration were observed for the corner-sharing oxygen atoms. While later theoretical studies showed that no true RUMs are present in the structure [59], the atomic displacement parameters extracted from diffraction data clearly demonstrate that transverse oxygen vibrations give rise to the observed NTE. While Sc_2_W_3_O_12_ remains orthorhombic at all temperatures, other compounds in this family undergo a symmetry-lowering displacive phase transition to a denser monoclinic polymorph at low temperatures (Figure 5b). In general, the orthorhombic Sc_2_W_3_O_12_ structure is classified as an NTE polymorph, while positive expansion has been reported for monoclinic phases.

The phonon density of state (DOS) for Sc_2_W_3_O_12_ has been determined from specific heat measurements. Like for ZrW_2_O_8_, very low energy phonons (~5 meV) with negative γ_i_ values, and a gap between the low and high energy phonons were observed [21]. Interestingly, a similar DOS distribution was found for Sc_2_Mo_3_O_12_, which adopts a monoclinic structure at low temperature. This suggests that the monoclinic structure has the potential to exhibit NTE as well. While the higher energy phonon contributions with positive γ_i_ values outweigh the effect of the low energy phonons with negative Grüneisen parameters at most temperatures, negative expansion of the b axis (4–60 K) and very small negative volume expansion (4–30 K, α_v_ = −3.7 × 10^−7^ K^−1^) were observed at very low temperatures [117].

#### 2.3.1. Substitution of Sc_2_W_3_O_12_

The scandium tungstate structure shows excellent tolerance towards ionic substitution of both metal sites. In most A_2_M_3_O_12_ compounds, the M site is occupied by Mo or W. In these cases, the A site can be substituted by any trivalent cation ranging in size from Al^3+^ (r_oct_ = 0.54 Å) to Ho^3+^ (r_oct_ = 0.90 Å). Ln_2_M_3_O_12_ compositions with larger lanthanides crystallize in structures that adopt higher coordination numbers (7 or 8) for the A-site. However, for solid solutions of two trivalent cations, significant amounts of larger Ln^3+^ can be incorporated into the scandium tungstate structure, as evidenced by reports of Er_2−x_Ce_x_W_3_O_12_ (x ≤ 0.4) [118], Er_2−x_Sm_x_W_3_O_12_ (x ≤ 0.5) [119], Er_2−x_Nd_x_W_3_O_12_ (x ≤ 0.5) [120], Er_2−x_Dy_x_W_3_O_12_ (x ≤ 0.7) [121], Y_2−x_Dy_x_W_3_O_12_ (x ≤ 1.0) [121], and Y_2−x_Nd_x_W_3_O_12_ (x ≤ 0.4) [122]. In addition, substitution with aliovalent cations has been achieved in AP_2_MO_12_ [123,124] and MgAM_3_O_12_ (A = Zr, Hf; M = Mo, W) [125,126,127]. AP_2_MO_12_ adopts the same structure as Sc_2_W_3_O_12_ with an ordered arrangement of P and M [124], while MgAM_3_O_12_ crystallizes in a different orthorhombic structure in space group Pnma or Pna2_1_ [125,126].

Many A_2_M_3_O_12_ compositions adopt a monoclinic structure at low temperatures, and transform to the orthorhombic Pnca phase upon heating [117]. The temperature for this phase transition is generally higher for molybdates than for tungstates, and increases with increasing electronegativity of the A-site cation [128]. This observation can be explained by the fact that oxygen-oxygen repulsive interactions make the denser monoclinic phase less favorable. More electronegative A-site elements reduce the partial charge on the oxygen atoms and thus the repulsive forces. However, shifts in the temperature or the complete absence of the phase transition for some mixed A-site compounds suggest that entropic factors also play a role. As the monoclinic phase generally displays positive expansion, this phase transition is undesirable for any potential uses of these NTE compounds in composites.

The expansion coefficient of the orthorhombic phase depends strongly on the identity of the A-site cation. As stated earlier, the A_2_M_3_O_12_ structure does not support RUMs, and distortion of the polyhedra occurs during the transverse vibrations of oxygen atoms. Larger A-site cations give rise to softer octahedra that distort more easily. As a result, expansion tends to become more negative with increasing size of A^3+^, reaching values of −7.0 × 10^−6^ K^−1^ in Y_2_W_3_O_12_ [129] and −9.3 × 10^−6^ K^−1^ in Y_2_Mo_3_O_12_. Additional slight decreases have been achieved in solid solutions of these compounds with larger lanthanides [118,119,120,121,122]. In contrast, Al_2_W_3_O_12_ has been reported to show low positive expansion with an α_l_ value of +2.2 × 10^−6^ K^−1^ [5]. Reports on expansion coefficients vary not only between α_l_ values extracted from variable temperature diffraction data and dilatometry, but also between different diffraction measurements. These discrepancies may in some cases arise from averaging over different temperature ranges, as the volume expansion of many A_2_M_3_O_12_ compounds is not completely linear. Nevertheless, the accumulated knowledge on the expansion behavior of different compositions can be used to design materials with desired α values, including zero expansion compounds. The first report of a solid solution with close to zero expansion around room temperature (InAlW_3_O_12_) was published in 1999 by Mary and Sleight [23]. More sophisticated solid solutions like Al_2x_(MgHf)_1−x_W_3_O_12_ have since been reported [130].

**Figure 6:**
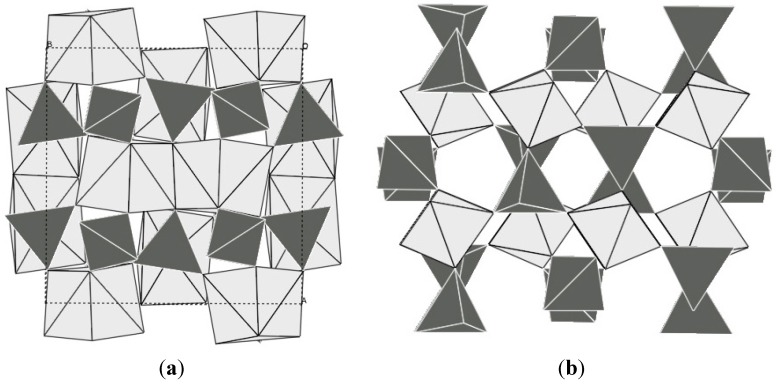
Crystal structures of (**a**) Pba2-Y_2_Mo_3_O_12_; (**b**) Pbcn-Y_2_Mo_3_O_12_; bright: YO_n_ polyhedral; dark: MoO_4_ tetrahedra. In the denser Pba2 polymorph, the coordination number of yttrium is increased, and the polyhedra share edges.

While the record negative expansion coefficients in the A_2_M_3_O_12_ family have been achieved with rare earth elements or the “pseudo-lanthanide” Y, it is necessary to point out that all of these compositions suffer from hygroscopicity. Under ambient conditions, a trihydrate is formed by absorption of moisture from the atmosphere [118,119,120,121,122,131,132]. This limits the usefulness of these compositions to temperatures above the dehydration point or sealed systems. In addition, several of these compounds could be on the borderline of thermodynamic stability of the Pnca-NTE polymorph, as it was shown that this structure is only thermodynamically stable above 823 K for Y_2_Mo_3_O_12_, and metastable with respect to a denser Pba2-polymorph at lower temperatures [133] (Figure 6).

#### 2.3.2. High Pressure Behavior

A number of A_2_M_3_O_12_ materials have been studied under pressure by diffraction and spectroscopic methods. Orthorhombic compounds tend to undergo a transition to the denser monoclinic polymorph at very low pressures (<0.6 GPa) [134,135,136,137,138]. Most compounds investigated *in situ* undergo at least one additional crystalline-to-crystalline phase transition [135,137,138,139,140,141,142,143,144,145,146,147]. Pressure-induced amorphization is observed at pressures ranging from 5 GPa to 20 GPa. Similar high pressure diffraction patterns were observed for Sc_2_W_3_O_12_ [136], Sc_2_Mo_3_O_12_ [135], Al_2_W_3_O_12_ [143], and Ga_2_Mo_3_O_12_ [146], but due to limited data quality, the structures have not been characterized.

### 2.4. Other NTE Oxides

**Figure 7:**
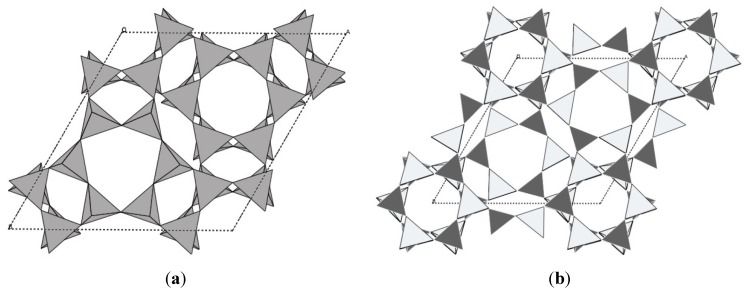
Crystal structures of (**a**) chabazite; (**b**) AlPO-17.

In addition to the ZrW_2_O_8_, ZrV_2_O_7_ and Sc_2_W_3_O_12_ families discussed so far, NTE due to transverse atomic vibrations has been observed in several other oxide structures. Many zeolites and zeolitic frameworks (AlPOs, GaPOs) exhibit this unusual property, which is not surprising considering that their structures are composed of corner-sharing tetrahedral networks [24,148]. In “normal” zeolites that contain a mixture of Si and Al, the presence of counter ions and water interferes with NTE, as the amount of empty space is reduced. This makes pure siliceous zeolites and group 13 phosphates better targets for studying NTE in zeolites. The magnitudes of NTE differ widely from close to zero in chabazite to the record of α_l_ = −11.7 × 10^−6^ K^−1^ in AlPO-17 [24,55,149] (Figure 7).

Another interesting example is found in Ag_2_O and Cu_2_O, in which the cations are linearly coordinated by two oxygens, and occupy the corners of the tetrahedral network of OM_4_ units. The overall structure consists of two independent, interpenetrating OM_4_ networks. NTE in Cu_2_O at low temperatures was first reported in 1985 [150], while this behavior was only documented in Ag_2_O in 2002 [13] EXAFS studies indicate significant distortion of the OM_4_ units, showing that a RUM model is not adequate to describe their expansion behavior [13,151]. A recent PDF study suggests that the distortion can lead to a shortening of the tetrahedral edge length and thus contribute to the NTE arising from transverse vibrations [43].

NTE has also been reported in materials with the delafossite structure [152,153,154], A^IV^_2_O(PO_4_)_2_ (A = Zr, Hf, U, Th) [155,156,157,158,159], ReO_3_ [160], NbOPO_4_ [161,162,163], NbVO_5_ [164], and TaVO_5_ [165]. While not an oxide, strong NTE in ScF_3_, which adopts the simple ReO_3_ structure, was reported by Greve *et al.* in 2010 [166]. This compound constitutes the first example of NTE over a wide temperature range in a purely octahedral framework.

### 2.5. Metal Cyanide Networks

Negative thermal expansion in metal cyanide frameworks was first reported for Zn(CN)_2_ by Williams *et al.* in 1997 [167]. This compound adopts a purely tetrahedral framework similar to silicates, but instead of a corner-sharing oxygen, the tetrahedra are connected by a diatomic cyanide linker. Fe[Co(CN)_6_] was the first purely octahedral metal cyanide network reported to display NTE in 2004 [58]. Its structure is related to the ReO_3_ structure by replacing the corner-sharing oxygen atoms of the octahedra by a diatomic cyanide bridge. Such frameworks can accommodate the same polyhedral rocking motions that cause NTE in the oxide families discussed so far, but the diatomic bridges give the structures more flexibility, which favors low frequency vibrational modes and increases the number of possible RUMs [168] The transverse vibrations of the CN linkers could be directly observed by pair distribution function analysis [15].

Metal cyanide frameworks quickly became the record holders for strongest NTE behavior with reports of α_l_ values as low as −33 × 10^−6^ K^−1^ for Cd(CN)_2_ [20], −40 × 10^−6^ K^−1^ for Zn_3_[Fe(CN)_6_]_2_, Fe_3_[Co(CN)_6_]_2_ and Co_3_[Co(CN)_6_]_2_ [169], and −48 × 10^−6^ K^−1^ for Mn_3_[Co(CN)_6_]_2_ [169]. It was also recognized that like in zeolites, the presence of guest molecules in the open frameworks interferes with the transverse vibrations of the linker groups, thus reducing the NTE coefficients [57]. Additional materials were synthesized, and composition dependent NTE was reported for a number of metal ions [18,170].

In 2008, metal cyanide networks set another record when colossal positive and negative (colossal: |α| ≥ 100 × 10^−6^ K^−1^) expansion behavior was observed in Ag_3_[Co(CN)_6_] along different unit cell axes [19]. The a axis of the trigonal unit cell expands with +130 × 10^−6^ K^−1^ ≤ α_a_ ≤ +150 × 10^−6^ K^−1^, while the c axis shows a similar magnitude of contraction with −130 × 10^−6^ K^−1^ ≤ α_c_ ≤ −120 × 10^−6^ K^−1^. Transverse vibrations of cyanide groups, which are observed in the structure, cannot account for the observed expansion behavior. The simultaneous observation of colossal positive and negative expansion points to the crystal structure as the source of this unusual behavior [171]. This material is composed of sheets of Co(CN)_6_ octahedra that are separated by layers of Ag^+^ ions. Each Ag^+^ is linearly coordinated by two cyanide groups, one from each adjacent layer. Ag···Ag contacts close to the sum of van der Waals radii are observed, which suggest argentophilic interactions. These Ag···Ag and the corresponding [Co(CN)_6_]···[Co(CN)_6_] contacts increase rapidly with temperature. In order to preserve the Co-CN-Ag-NC-Co bridges in the material, the strong expansion along the a axis must be coupled to equally strong contraction along the c axis, which has been described as similar to “garden lattice fencing”. It was later established that the argentophilic interactions between silver atoms are the major driving force for the observed colossal expansion behavior, as isostructural D_3_[Co(CN)_6_] did not display colossal behavior [172]. However, strong metallophilic interactions can reduce expansion coefficients [44].

## 3. Composites

One of the reasons why NTE materials have attracted significant interest from the science and engineering communities lies in their potential applications as fillers in controlled thermal expansion composites. Mismatches in thermal expansion of device components have long been recognized as a serious problem, whether in the expansion of road surfaces, railroad tracks and bridges, or in thin films where lattice matching at deposition and use temperatures is important. Precision positioning is critical in electronic devices, and nanotechnology only amplifies the level of control necessary for proper function.

In most modern applications, very specific materials’ properties are required, which may include conductivity, magnetic and optical properties, hardness, ductility or more. This can make it difficult to achieve zero expansion or match the expansion coefficient of another device component. In such cases, use of composites is an attractive alternative, which can allow preservation of desirable material properties while modifying unfavorable ones or adding new, advantageous ones. NTE materials can theoretically be used to reduce or offset the expansion of any other material through preparation of controlled thermal expansion composites. In practice, the preparation of high quality composites poses a number of interesting engineering challenges.

### 3.1. Desirable Properties of Filler Materials

The best filler material for any controlled expansion composite is the least expensive compound that will achieve the desired reduction in expansion and can easily be prepared in the necessary quantities. In addition, stability under processing and use conditions, and compatibility with other system components, is necessary. *In situ* phase formation may aid in achieving homogeneous composites, alternatively, small particle sizes will improve mixing. Scaling up processes to industrial scale will likely bring additional challenges. Materials with isotropic NTE offer the advantage that filler particle orientation does not affect expansion, making isotropic behavior desirable in many cases. However, if the expansion of a highly oriented matrix or device needs to be compensated, anisotropic NTE may be preferable.

The exact optimum properties of a filler material can change depending on the exact nature of the composite. For example, compatibility will depend on the identity and properties of other device components. Similarly, the targeted application for a device will often dictate processing and conditions (temperature, pressure, atmosphere/environment *etc.*). Nevertheless, there are certain known properties of NTE materials that could impede their usefulness in controlled thermal expansion composites, which will be discussed in the following section.

### 3.2. Potential Problems with Known NTE Filler Materials

With the exception of zeolitic materials, all NTE materials discussed in this review contain transition metals, which generally increases the price of the starting materials. For high end applications where other device components or manufacturing processes already have a significant cost, the added expense of small amounts of NTE materials becomes negligible. However, for mass production of cheaper devices, the NTE filler cost may prove prohibitive. Zr, V, Mo and W based starting materials are significantly more expensive than zeolites, and many Sc_2_W_3_O_12_-type compounds that show NTE around room temperature contain expensive lanthanides or pseudo-lanthanides. Commercial availability is another important consideration. Zeolites are readily available, but ZrW_2_O_8_ is the only NTE material discussed in Section 2.1 through Section 2.3 that can be purchased. The commercially available ZrW_2_O_8_ powder has a median particle size of 15-20 μm, and may contain small ZrO_2_ and WO_3_ impurities.

The ease of preparation of NTE fillers depends on their thermodynamic stability and whether particle size control is necessary. Most compounds in the ZrV_2_O_7_ and Sc_2_W_3_O_12_ families are thermodynamically stable, and can be obtained by traditional ceramic methods [1,5]. In contrast, the ZrW_2_O_8_-type materials are metastable at room temperature, requiring rapid quenching and thus limiting batch sizes for traditional solid state synthesis [34,71]. Preparation of significant quantities of ZrMo_2_O_8_ or HfMo_2_O_8_ by high temperature approaches may not be feasible. In addition, P_2_O_5_, V_2_O_5_, MoO_3_ and WO_3_ show considerable volatility at high temperatures, making extended heating of binary oxide starting mixtures unfavorable. Solution based routes to all families of NTE compounds have been reported, which offer the added advantage of particle size control [173,174]. However, synthesis conditions must be carefully optimized to ensure preparation of stoichiometric, homogeneous NTE compounds, especially for complex solid solutions. For the ZrW_2_O_8_ family, low temperature routes require topotactic recrystallization at a temperature where the compounds are metastable, which further complicates the preparation of phase pure samples, especially for the molybdates [69]. AM_2_O_8_ compounds and other NTE materials with an upper limit of thermal stability are not suitable for ceramic composites that require sintering at high temperature.

The temperature range over which a material displays NTE is important. For most applications, this range should include room temperature, and a wider range of NTE can be considered beneficial. This makes several zeolites, the ZrV_2_O_7_ family and Sc_2_W_3_O_12_-type materials with smaller A^3+^ cation less attractive.

A major drawback of many NTE materials is their instability under moderate pressure. Composite preparation has to avoid pressures at which the filler irreversibly transforms to a different phase. In addition, pressure-induced phase transitions can cause problems during thermal cycling of composites if localized pressures on individual filler particles exceed the transition pressure. ZrW_2_O_8_ and HfW_2_O_8_ show the most detrimental behavior in this respect, with irreversible phase transitions at 0.2 and 0.6 GPa, respectively [84,89]. While many orthorhombic A_2_M_3_O_12_ compounds transform to a denser monoclinic phase at 0.3 to 0.7 GPa as well [135,136,143], this transition is reversible upon decompression. Zr_2_WP_2_O_12_ transforms to the denser monoclinic polymorph as well, but only above 1.4 GPa [145].

Another problem arises from the instability of some NTE compounds under ambient conditions unless they are used in a sealed system. A_2_M_3_O_12_ compositions are very hydroscopic when A is a lanthanide or yttrium, forming a trihydrate A_2_M_3_O_12_·3H_2_O within minutes of exposure to atmospheric moisture. While the water can be removed by heating, repeated hydration and dehydration is likely to accelerate composite deterioration. Solid solutions Zr_1−x_Hf_x_Mo_2−y_W_y_O_8_ with 30 to 90% tungsten content also incorporate water into their crystal structures [175,176], although the absorption is slower and does not lead to a significant change in crystal structure. Recently, this autohydration behavior was also observed in nanosized ZrW_2_O_8_ [177], while micron sized particles required hydrothermal treatment at 180 °C to force water into the structure [175].

Lastly, compatibility and mixing of filler and matrix are important for the preparation of composites. While other components in ceramic composites are usually compatible with NTE fillers, reactivity towards metals has been reported [49], and poor interaction between as-prepared filler particles and matrix has been observed in polymer composites [178,179,180].

### 3.3. Literature Reports on Composites with NTE Fillers

To date, literature reports on composites using the NTE fillers discussed in this review article are sparse. Zeolitic materials have not been utilized in attempts to reduce other materials’ expansion, despite their commercial availability and lower cost. This may be related to the fact that their expansion behavior is strongly influenced by guest molecules, making their behavior in composites less predictable. Interestingly, the NTE observed in many zeolites has been reported as a problem, as their shrinkage leads to crack formation and delamination between zeolite membranes and alumina supports [181,182]. Recently, use of a zeolite-based support instead of alumina was proposed to overcome this expansion mismatch [183].

With two exceptions, NTE composite research has focused on ZrW_2_O_8_. A ceramic Fe_x_Sc_2−x_Mo_3_O_12_/MoO_3_ composite with close to zero thermal expansion was prepared by melt reaction [184]. The final composite contained significant void space and was brittle, which was attributed to evaporation of MoO_3_. In addition, a magnesium composite containing 20 vol% Zr_2_WP_2_O_12_ was reported [185]. The overall expansion coefficient was only slightly reduced.

ZrW_2_O_8_ has been incorporated into metal, ceramic and polymer composites. Most of the metal composite work focused on Cu composites [49,50,186,187,188,189,190]. Cu is used as a heat sink in microelectronics, and copper composites that match the expansion coefficient of Si (4 × 10^−6^ K^−1^) or Al_2_O_3_ (7 × 10^−6^ K^−1^) could find widespread applications [49]. However, in all cases, formation of significant amounts of orthorhombic γ-ZrW_2_O_8_ was observed during thermal cycling. It was found that the copper matrix exerts a pressure of ~0.45 GPa on the filler particles [187,189], which is high enough to induce the cubic to orthorhombic phase transition. Attempts to reduce the local stress through precoating of particles with Cu did not succeed in suppressing the transformation [190]. It should be possible to overcome this problem by using HfW_2_O_8_ as a filler instead of ZrW_2_O_8_, however, no such attempts have been reported to date. An exploratory investigation on a ZrW_2_O_8_/low expansion steel composite (α_steel_ = 1.5 × 10^−6^ K^−1^) still observed formation of γ-ZrW_2_O_8_, clearly indicating that the application of ZrW_2_O_8_ will be limited to environments that will not exert significant pressure on the filler particles [191].

The exploratory synthesis of ZrW_2_O_8_/SiO_2_ [192] and ZrW_2_O_8_/cement composites [193] have been reported. However, most ZrW_2_O_8_/ceramic composites have used ZrO_2_ [51,52,194,195,196,197,198,199,200] or Zr_2_WP_2_O_12_ [201,202,203] as the second component. Due to the similarity of the two components, good compatibility was observed. Formation of dense ceramic bodies (up to 95% dense) was achieved by addition of small amounts of Al_2_O_3_ [194,195], and control of thermal expansion ranging from negative to positive values was achieved for ZrO_2_, while all ZrW_2_O_8_/Zr_2_WP_2_O_12_ mixtures exhibited negative expansion. The α- to β-transition of ZrW_2_O_8_ was suppressed for composites with 75% Zr_2_WP_2_O_12_.

ZrW_2_O_8_ has been incorporated into several polymer systems, including phenolic resins [204], epoxy resins [205] and polyimides [178,179,180]. In contrast to oxide ceramics, ZrW_2_O_8_ is not readily compatible with most polymer systems, and surface modification of filler particles is necessary to achieve good interaction with the matrix [178,179,205]. Significant reductions in expansion have been observed in all cases, ranging from 30% reduction for phenolic resins with 52 vol% and polyimides with 22 vol% filler to 60% reduction in epoxy composites for 40 vol% loading. Significant particle agglomeration was observed when large filler particles were used. In addition, large particles are prone to settling at the bottom of any polymer composite films during film formation. Nanoparticles can be used to overcome this problem [178,179], however, a compromise between particle size and kinetics of the recently reported autohydration [177] will have to be found. Optimization of processing conditions like the use of reprecipitation blending reported for polyimide composites may allow formation of homogeneous ZrW_2_O_8_/polymer composites with intermediate particle size [179].

## 4. Conclusions

Negative thermal expansion has been established as a specialized field of research since the mid 1990s. New materials belonging to previously identified families of compounds, new families of NTE materials, and new insights into mechanisms are added to the literature every year. The initially predicted widespread use of NTE materials as fillers in a variety of controlled thermal expansion composites has not yet been achieved, but some promising preliminary results on ZrW_2_O_8_/ZrO_2_ and ZrW_2_O_8_/polymer systems have been reported. Exploration of other filler materials that are less pressure sensitive or do not autohydrate under ambient conditions will be necessary to further the applications of NTE fillers, and will likely lead to interesting application as the field matures.
